# Specialized cattle farming in the Neolithic Rhine-Meuse Delta: Results from zooarchaeological and stable isotope (δ^18^O, δ^13^C, δ^15^N) analyses

**DOI:** 10.1371/journal.pone.0240464

**Published:** 2020-10-21

**Authors:** Safoora Kamjan, Rosalind E. Gillis, Canan Çakırlar, Daan C. M. Raemaekers

**Affiliations:** 1 Groningen Institute of Archaeology, University of Groningen, Groningen, Netherlands; 2 Interdisciplinary Center for Archaeology and Evolution of Human Behaviour, University of Algarve, Faro, Portugal; University at Buffalo - The State University of New York, UNITED STATES

## Abstract

Schipluiden (3630–3380 cal BC), the earliest known year-round settlement in the Rhine-Meuse Delta in the Netherlands, is a key site for addressing the nature of Neolithic subsistence in the wetlands of northwestern Europe. A preliminary zooarchaeological study suggested that cattle husbandry was a major activity at Schipluiden. In contrast, stable carbon and nitrogen isotope analyses of human remains from the site indicated a marine-oriented diet, implying that the Mesolithic-Neolithic dietary transition continued well into the mid-4^th^ Millennium BC in this region. Here, we re-investigate the role and nature of cattle husbandry at Neolithic Schipluiden using mortality profiles and stable isotope analysis (δ^18^O, δ^13^C, δ^15^N) of animal bone collagen and tooth enamel. The age-at-death analysis suggests that cattle were managed for both meat and milk production. The δ^18^O and δ^13^C analysis of tooth enamel provide evidence that calving spread over five-and-a-half-months, which would have led to a longer availability of milk throughout the year. Cattle were grazing in open, marshy environments near the site and winter foddering was practiced occasionally. The faunal isotopic data also reveal that the high ^15^N in human bone collagen is more likely to signal the consumption of products from cattle that grazed on ^15^N-enriched salt marsh plants around the site, rather than a marine-oriented diet. This undermines the previous interpretation of the dietary practices at Schipluiden by showing that human diet in mid-4^th^ millennium BC Rhine-Meuse area was fully “Neolithic”, based primarily on products from domesticates, especially cattle, with some input from wild terrestrial and aquatic resources available in their surroundings, contrary to what has been proposed before. Collating these results demonstrates a high level of investment in cattle husbandry, highlighting the social and economic importance of cattle at the lower Rhine-Meuse Delta during the 4^th^ millennium BC.

## Introduction

The domestication of cattle (*Bos taurus*) and its subsequent spread into Europe caused unprecedented economic, biological, and social transformations in both animal and human history [1–4]. Management of domestic cattle made important protein resources like meat and dairy more accessible [5,6], leading to a rapid and complete shift in human diet from marine to terrestrial protein as reported in Britain and Iberia with the onset of farming [7–10]. Manure could be collected to fertilize crops and to be used as fuel [11,12]. Using cattle for ploughing and traction improved cultivation and transportation [13,14]. Maintaining these benefits in diverse and changing environments and social contexts necessitated increased human influence on cattle mortality, reproductive behavior, diet, and mobility [6,15–17]. The major investment in managing this animal strengthened its social and economic importance in addition to its nutritional value [4,18–20].

**Fig 1:**
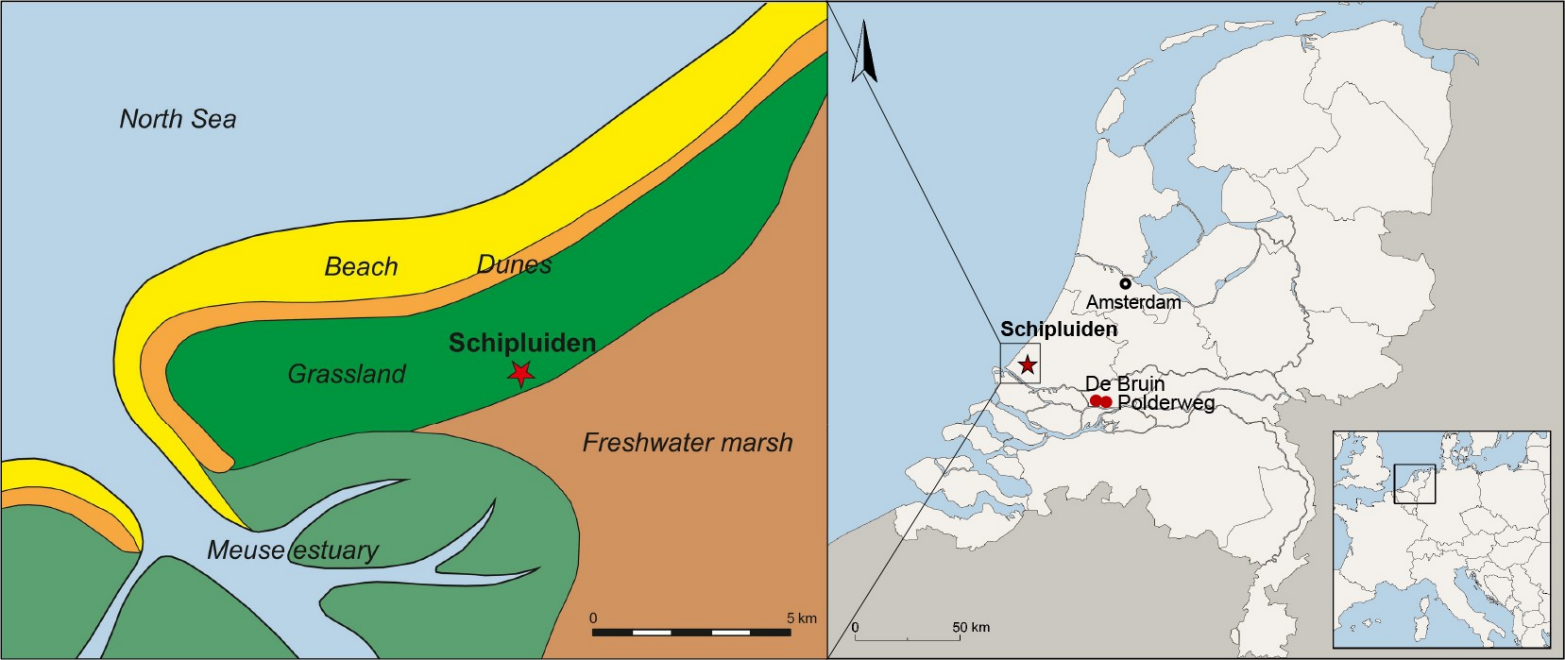
Left: Palaeogeographic map of the study area depicting the region at the beginning of the 4^th^ millennium BC (after [32], Fig 14). Right: Location of Schipluiden, De Bruin, and Polderweg on a modern geographic map of the Netherlands (ArchGIS online basemaps used for producing the map. drawing S. Tiebackx and S. Boersma, Groningen Institute of Archaeology).

In Neolithic temperate Europe, the dominance of cattle in zooarchaeological assemblages and symbolism has highlighted the multifaceted dimensions of human-cattle relationships in this region and been the focus of a wide range of studies over the last decades [18,21,22]. In some parts of Europe, such as the Rhine-Meuse Delta in the Netherlands, the significance of cattle husbandry in the Neolithic has not been thoroughly explored [22]. Debates about the onset of the Neolithic in the Dutch Delta, specifically how and when animal husbandry began, continue to be inconclusive due to fragmentary zooarchaeological data, small sample sizes, and scarce ancient DNA (aDNA) and stable isotope analysis [23]. The available stable isotope data indicate a freshwater-based diet for the inland Mesolithic population from the sites of Hardinxveld-Giessendam-Polderweg (5450–5050 BC) and Hardinxveld-Giessendam-De Bruin (5250–4500 BC) located in the Rhine-Meuse Delta [24]. In comparison, Swifterbant sites (transitional Mesolithic-Neolithic; ca. 4300–4000 BC) in the northeast of the Flevolands Polders of the Netherlands appear to have a mixed diet (terrestrial and freshwater) [25,26]. Among the Rhine-Meuse Delta Mesolithic settlements, cattle remains are absent or scarce and they cannot be readily distinguished from aurochs based on their morphology, isotopes, or mortality patterns [22]. Cattle remains are infrequent in the Swifterbant sites and their domestication remains a subject of debate [27]. Year-round settlements with ample evidence for agriculture and animal husbandry appear along the Rhine-Meuse Delta after 4000 BC [28,29]. Among these so-called Hazendonk group sites, Schipluiden (Fig 1) is of particular interest because of its well-preserved and documented archaeological record [30]. The significance of cattle in the subsistence and symbolic activities of the Schipluiden community is highlighted by conventional zooarchaeological analysis [31]. However, when isotopic compositions of human bone collagen from the site suggested significant protein contribution from marine resources [24,25], the role of cattle husbandry in the region in subsequent research was dismissed (e.g. [22]).

Here, our objective is to redress this deficiency by investigating cattle husbandry at Schipluiden in detail, through zooarchaeological and stable isotope analyses. Using long bone fusion and tooth wear stages, we analyse cattle mortality profiles to identify slaughter management practices. We use the sequential analysis of stable oxygen (δ^18^O) and carbon (δ^13^C) isotope values in cattle tooth enamel to investigate cattle diet and calving season at the site. To infer cattle pasture usage and foddering practices, we use stable carbon (δ^13^C) and nitrogen (δ^15^N) isotope analysis of bone collagen. Furthermore, we explore the role of terrestrial protein in the human diet by integrating our isotopic dataset from the Schipluiden animal bone collagen with previously published isotopic data from human remains recovered from Schipluiden and the Late Mesolithic sites of Hardinxveld-Giessendam-Polderweg (hereafter Polderweg) and Hardinxveld-Giessendam-De Bruin (hereafter De Bruin), which provides us with a glimpse into the Mesolithic dietary patterns in the region [33,34].

This research represents the first integrated zooarchaeological and stable isotopic study on Neolithic faunal remains in the Dutch Delta and adds valuable information to the existing albeit scarce isotopic and faunal datasets available for the region to understand human-cattle relationships. By providing a substantial “faunal baseline” for the stable isotopic proxies of human palaeodiet in Schipluiden, it fine-tunes the earlier interpretations on human diet shift with the onset of farming in the Dutch Wetlands.

### The site

Schipluiden is situated on a coastal dune bordered to the south by the Rhine-Meuse estuary in the western part of the Netherlands [30]. Excavations in 2003 revealed three Neolithic occupational phases, separated by transitional mixed depositions, encompassing about 200 years in total. Radiocarbon analysis dates the occupation to between 3630–3380 cal BC [30]. The wealth of archaeological remains allowed detailed studies by specialists, published in an edited volume on the site [30]. Palynological studies indicate that Schipluiden was situated on a treeless dune within an open C_3_ vegetation, approximately 3 km from the sea at the time of occupation. This area was under the direct influence of the sea where salt marsh clay was being deposited. Freshwater marshes with patches of alder carr were to be found c.1 km inland [35]. The brackish environment began to develop into a freshwater environment towards the later phases of the occupation [35]. Studies on zooarchaeological remains (mammals, birds, fish, and mollusks), however, showed no major diachronic shift in faunal composition, which represents both freshwater and brackish environments throughout the occupation [31,36–38].

The human subsistence strategies have been based on the exploitation of a wide range of natural resources, supplemented by crop cultivation and animal husbandry, which Louwe Kooijmans and Jongste viewed as an “extended broad-spectrum economy” [30]. Archaeobotanical analysis suggests the exploitation of wild food plants from the surrounding dune along with local cultivation of emmer (*Triticum dicoccom*) and naked barley (*Hordeum vulgare* var. *nudum*) [39]. Among a total of 7750 hand-collected mammal remains, domestic cattle were the most numerous (comprising 42% of the number of finds (Nf)), suggesting cattle were the most important livestock in terms of meat yield [31]. A broad, fence-enclosed trampled zone around the settlement contained cow pats, confirming the presence of cattle in the vicinity of the site [40,41]. Sheep and goat remains were, notably, absent. Suid (*Sus* cf. *scrofa*) and red deer (*Cervus elaphus*) were the most commonly hunted species. Fur-bearing animals, namely beaver (*Castor fiber*), otter (*Lutra lutra*), and wildcat (*Felis silvestris*) were identified in low numbers. Sea mammals, such as common seal (*Phoca vitulina*) and bottlenose dolphin (*Tursiops truncatus*), made a very small contribution to the mammal assemblage. [31]. Waterfowl and duck (Anatidae) were the most common among the avifauna species [36]. The ichthyoarchaeological study highlighted the importance of fishing, with three migratory species–eel (*Anguilla anguilla*), flounder (*Pleuronectes flesus*), and Atlantic sturgeon (*Acipenser sturio*)–comprising 97% of the identified fish remains. Freshwater and marine fish were rare (c.3%) [37]. A noteworthy animal-related feature is a pit (Feature 12–48) that contained three cattle skulls and one heavily fragmented dog (*Canis familiaris*) skull [40]. The depositional characteristics of the pit were interpreted as a single event reflecting a deliberate activity [31].

A palaeodietary study on the human remains from Schipluiden suggested that the exploitation of marine resources was substantial, based on elevated δ^15^N and low δ^13^C values in eight samples of human bone collagen and the associated zooarchaeological evidence for fishing and fowling [24,25]. These studies did not integrate an isotopic faunal baseline from Schipluiden to further substantiate their claims. This has resulted in the researchers downplaying the importance of agropastoral activities at the site and suggest a marginal role for terrestrial food resources, in particular from cattle, in the Neolithic diet [42].

### Theoretical principles

Reconstructing the age-at-death and the sex ratio of livestock in the zooarchaeological record is mostly reliable through standardized observations on tooth eruption and wear stages, as well as biometric data. These methods enable estimating the relative abundance of smaller females and larger males in the adult population and the modalities of slaughtering management which are associated with animal exploitation strategies [6,43]. Careful recovery methods, methodological developments in reconstructing higher resolution kill-off patterns [44], and revised subsistence models for early livestock management [6,45] have recently revealed that Neolithic animal husbandry strategies depend on cultural and environmental parameters as well as the biology of the animals.

Investigating cattle birth seasonality through modeling of δ^18^O values provides additional insights into the length of calving season, which determines the duration of milk availability for human exploitation [15]. Cattle are physiologically able to breed throughout the year, but environmental (nutrition and climate) and physiological parameters are central factors that determine the calving season [46]. In temperate Europe, free-ranging cattle give birth from late winter to late spring, when fresh grass is abundant [47,48]. Physiological acclimatization to new environments and interference by farmers in the form of technical practices can alter birth seasonality [46,49]. Year-round availability of fresh grass or fodder supplementation can extend the birthing season leading to a longer availability of milk [46].

The birth season of prehistoric animals has been widely investigated through looking at intra-tooth variation in oxygen isotope ratios (δ^18^O) of sequentially sampled tooth enamel bioapatite [46,50–52]. In temperate Europe, the oxygen isotope composition in mammalian skeletal bioapatite is linked to body water [53,54] and correlated with seasonal air temperature. The δ^18^O of meteoric water reaches its highest value in summertime and decreases in the winter [55]. Skeletal bioapatite archives these values, which can be used to estimate the seasonality of birth in a herd. These values are recorded in tooth enamel during mineralization and do not change throughout the animal’s lifetime [15]. Because the timing of tooth growth is fixed within individual species, using the position in the tooth crown where the lowest and highest δ^18^O values are measured (*X*_*0*_) relative to the tooth enamel–root junction (erj), the inter-individual variability of the birth season within the sampled population can be measured [50]. To eliminate the effects of variability in tooth size, the obtained data is normalized to the periodic cycle of δ^18^O values (tooth length formed over a year, or *X*) and modeled based on the equation proposed in [50]. We use the Pearson’s correlation coefficient (Pearson’s *r*) to measure the proximity between the calculated (modeled) and measured δ^18^O sequence.

Stable carbon (δ^13^C) isotope values measured in tooth enamel bioapatite are a proxy for the animal’s total diet [56,57], while δ^18^O values provide the seasonal framework to interpret the results. Sequential sampling of high crown herbivore molars can be used to investigate diet during tooth development at an individual scale as well as the seasonal variation of animal diet, grazing environment, and foddering practices [58]. Pre-industrial C_3_ plants, the dominant plant type in Europe, have δ^13^C values between –27.5‰ to –23.5‰ (−25‰ on average) when growing in open environments [59]. Considering a +14.1‰ enrichment of ^13^C between tooth enamel bioapatite of consumers and their diet [60], all-year-round grazing in open environments would result in δ^13^C values of about –11‰ in bioapatite. Plants growing in closed forests can yield δ^13^C values as low as −31‰ due to recycling of ^13^C-depleted CO_2_ and reduced photosynthesis due to low light levels, known as the *canopy effect* [61,62]. Therefore, woodland grazing and foddering would be evident by low δ^13^C values, depending on the degree of canopy density [61].

Stable carbon and nitrogen isotope compositions of bone collagen are widely used to reconstruct past human and animal dietary patterns (e.g. [7,8,57,63]). The carbon isotope (δ^13^C) value reflects the average δ^13^C values of the dietary protein during the last years of an individual's life, whereas the stable nitrogen (δ^15^N) values indicate the protein intake level of an individual in a food web [64]. The enrichments from dietary δ^13^C and δ^15^N values and herbivores bone collagen δ^13^C and δ^15^N are about 5.1‰ and 3.5‰ respectively [57,65]. In carnivores, the enrichment in δ^13^C values is about 1‰ [66]. In animal bone collagen, the vegetation composition [67] and the contribution of marine or terrestrial protein to their diet [57] can influence the isotopic values and the enrichment levels. Year-round grazing on C_3_ plants in open environments would lead to δ^13^C values of –22.5‰ to –18.5‰ (–20‰ on average) in herbivores’ bone collagen, considering a 5.1‰ spacing between diet and collagen δ^13^C [57]. Values below –22.5‰ in collagen can be considered as the key value for grazing in open areas and an indication of resources from a closed-canopy environment [68]. Contribution of resources from marine/estuarine environments leads to elevated δ^13^C and δ^15^N values in bone collagen, whereas freshwater/riverine components will be reflected in low δ^13^C values compared to the terrestrial ecosystems [69,70]. Saltmarsh grazing is proved to elevate the δ^15^N values significantly (approximately 3‰) compared to other terrestrial resources as a result of salinity and sea-spray effect [71]. Therefore, the stable isotopic values measured in the animal bone collagen are expected to reflect the grazing environment of cattle and serve as a baseline to discuss the interpretations provided by the previous study on human palaeodiet at the site [24,25].

In human palaeodietary studies, faunal isotopic reference baselines are used to distinguish between terrestrial versus marine protein consumption. Overall, in human bone collagen δ^13^C values of ca. –12 ± 1‰ are representative of a diet with 100% marine protein component, whereas δ^13^C values of ca. –20 ± 1‰ mirrors a 100% terrestrial-based diet [72]. The values that fall in between these numbers would be interpreted as a diet composed of both marine and terrestrial sources.

## Material and methods

Study and sampling of the faunal remains were conducted under the permit no. 2019–10, which complied with all relevant regulations. This permit is granted by the Zuid-Holland Provincial Archaeological Depot in Alphen aan den Rijn, Netherlands, where the assemblage is stored.

To reconstruct the cattle mortality profile, we used mandibles with teeth embedded and loose lower teeth (NISP = 138) recovered through hand collecting and wet-sieving (4mm). We distinguished loose first (M1) and second (M2) mandibular molars based on the cervical length (CervL) relative to the anterior width (WA) [73]. We used the same measurements from 41 intact M1 and M2 embedded in the mandible from the assemblage as the reference data-set (S1 Table in S1 File). Using this reference, we were able to establish that 15 of the loose molars are M1 and the remaining 15 are M2 (S1 Fig in S1 File). We recorded tooth eruption, wear, and replacement stages described by Grant [74] and interpreted the stages following Legge [45] (S2 Table in S1 File). Legge’s stages were used because they have proved more accurate than other methods, such as Higham [75], and more consistent with post-cranial data [76]. We used the R code described in Gerbault et al. [44], adapted for Legge’s stages [45], to generate the mortality profile. This approach uses Bayesian statistics, in particular the Dirichlet distribution, to produce simulations based on the original age-at-death frequency distribution and it generates credible intervals for each age class.

Given that the fusion of long bones occurs within a certain age range [77], we used the fusion stages of 321 cattle long bones recorded in Zeiler [31] as another proxy for investigating the age-at-death in the assemblage. To estimate the sex ratios represented in the mortality profile, we used 16 cattle metacarpal distal breadth (Bd) measurements [31]. Since artiodactyls are sexually dimorphic, such data can be used as a proxy for the sex ratio of the animals which survived past the fusion stage for this bone (24–36 months, following Reitz et al. [77], Tables 3.5; page 72) with the smaller measurements representing female individuals.

For the sequential sampling of tooth enamel, we selected only the lower third molars to make the data comparable with previous studies on cattle birth seasonality [15,68]. We assessed the domestic status of cattle using the ratio of cervical length (CervL) to width of anterior (WA) mandibular third molars (n = 18) in comparison with wild bovine measurements from Denmark [78]. These results are reported in the S3 Table and S2 Fig in S1 File. All the molar measurements are smaller than published aurochs’ measurements, indicating that they probably belong to domestic individuals. We selected eight third molars from different individuals with relatively similar wear stages for sequential sampling (Legge’s stage 6) [45] (S4 Table in S1 File).

After cleaning the surface of the tooth, sequential sampling of the enamel powder was conducted on the anterior lobe by drilling with a diamond bur, perpendicular to the tooth growth axis. Approximately, 7–10 mg of enamel powder was taken for each sample. Depending on the length of the tooth, 16–22 samples were taken from each tooth, starting from the crown and continuing to the enamel*–*root junction. A total of 151 samples were obtained from the eight cattle molars. The distance of each sample from the tooth enamel*–*root junction was recorded. Sampling was undertaken at the Centre for Isotope Research (CIO), University of Groningen, following the protocol proposed by Balasse [58]. Stable isotope analyses were conducted in the Service de Spectrométrie de Masse Isotopique du Muséum national d'Histoire naturelle (SSMIM) in Paris. Methods used for stable oxygen and carbon isotope analysis are described in S1 Text in S1 File.

For bone collagen extraction, we selected 45 faunal specimens from the cultural layers at the site. The samples represent wild and domestic species: cattle (n = 20), red deer (n = 6), dog (n = 3), suids (n = 6), sturgeon (n = 4), flounders (Pleuronectidae, n = 2), eel (n = 1), pike (*Esox lucius*, n = 1), and thinlip mullet (*Liza ramada*, n = 2). All individuals are adults based on their morphology and/or skeletal fusion. Among the cattle remains, we only selected the elements which are smaller than the wild female aurochs from Denmark to ensure that only domesticated animals were analyzed (Measurements reported in Zeiler [31]. Collagen extraction and isotopic measurement took place at the Center for Isotope Research (CIO), University of Groningen. Methods used for stable carbon and nitrogen isotope analysis are described in S2 Text in S1 File.

## Results

### Cattle mortality profile

**Fig 2:**
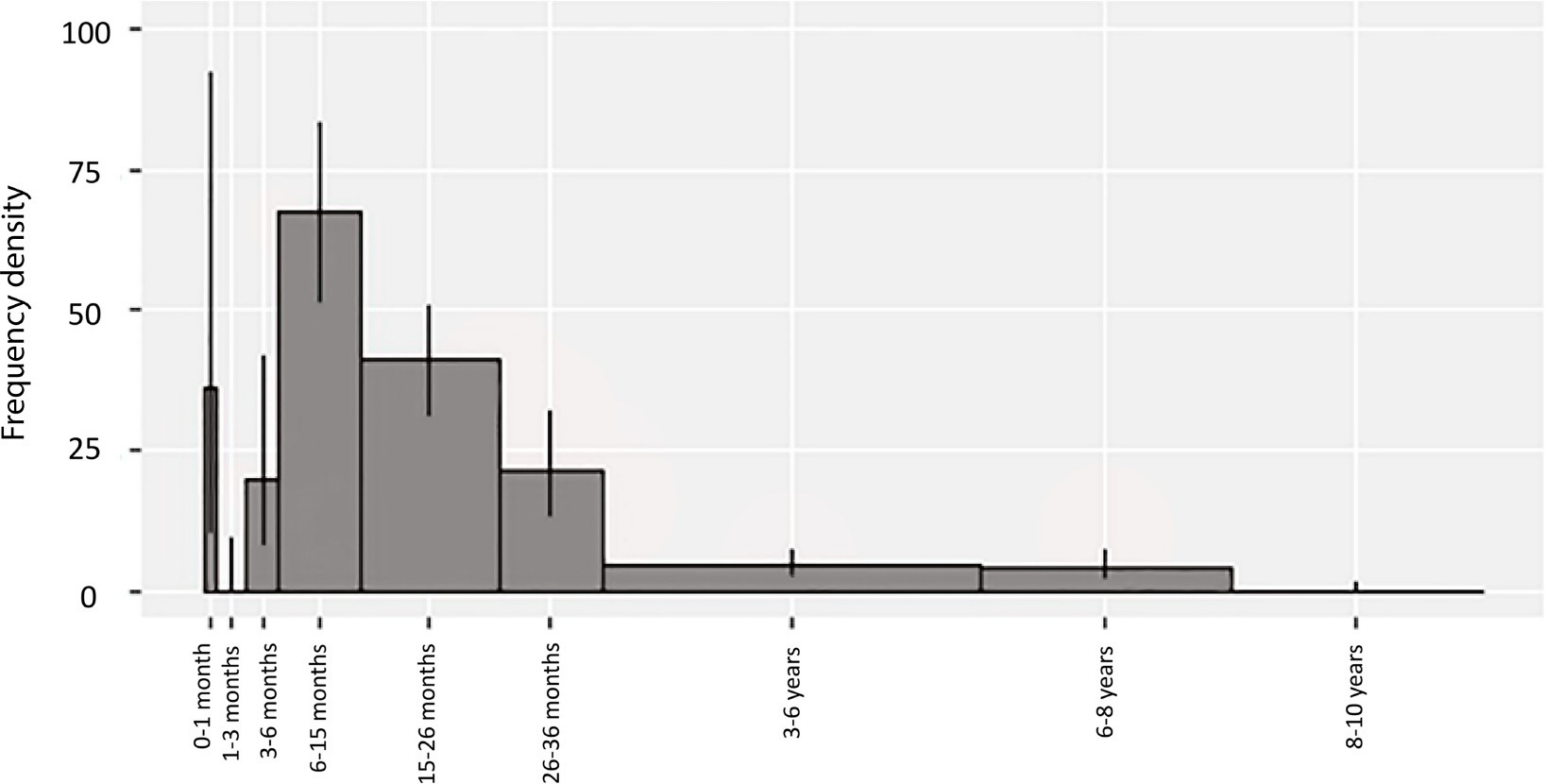
Cattle mortality profile from Schipluiden based on tooth eruption and wear stages recorded following Legge [45] and Grant [74]. (NISP = 138). Statistical method after Gerbault et al. [44].

The Schipluiden cattle mortality profile based on dental remains is presented in Fig 2, with error bars representing 95% credible intervals of the frequency density for each age class. The mortality in age class 0–1 month has a high-frequency density but also a large credible interval. The mortality profile has the largest peak in the age group 6–15 months (NISP = 45). Young adults between 15–26 months and 26–36 months also make a large contribution to the mortality profile (NISP = 45 and NISP = 18, respectively). Individuals older than 3 years are represented (3–6 years: NISP = 14; 6–8 years: NISP = 8). Individuals older than 8 years are not attested in the assemblage.

**Fig 3:**
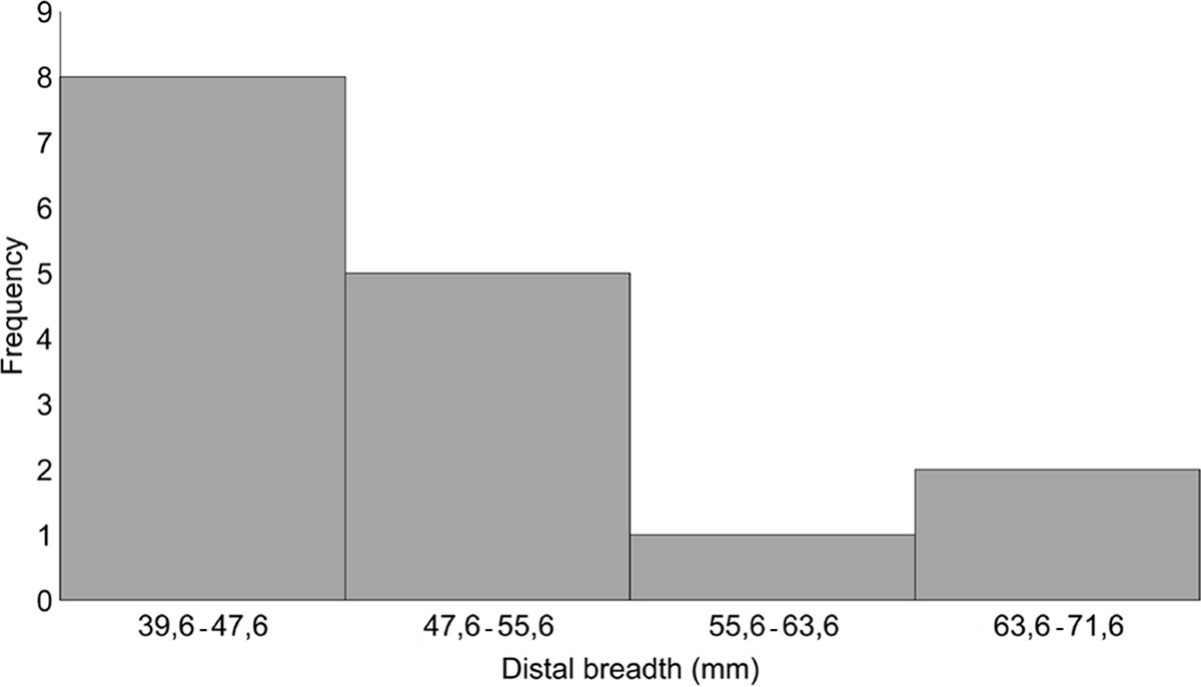
Cattle metacarpal distal breadth from Schipluiden (n = 16). Data from Zeiler [31].

The distal breadth (Bd) of 16 cattle metacarpals are between 40 and 71mm, showing a bimodal distribution. The peak of smaller metacarpals (12 out of 16) reflects the higher number of mature females slaughtered (Fig 3).

### The δ^13^C and δ^18^O measurements of enamel bioapatite

**Fig 4:**
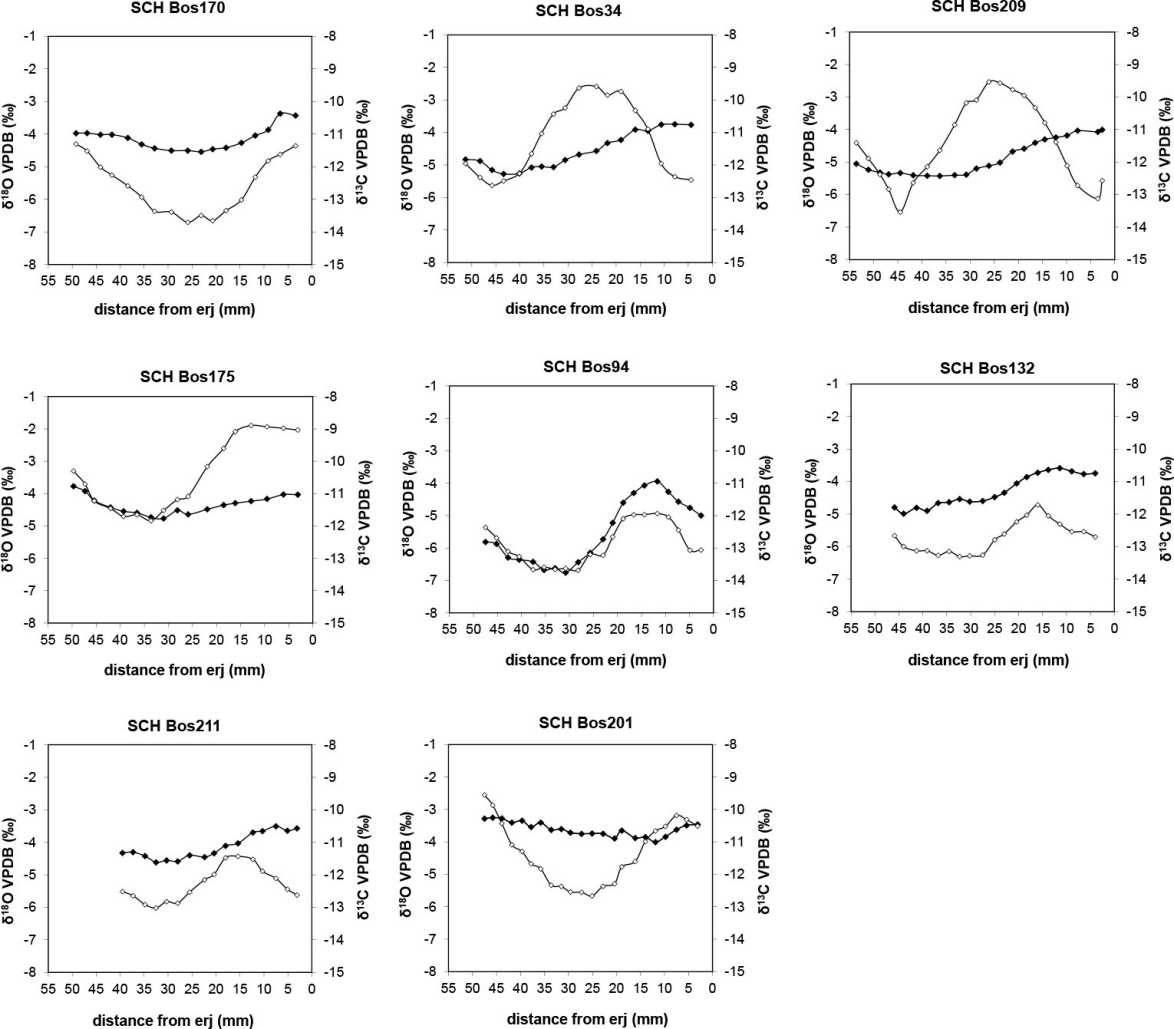
Results from stable oxygen (δ^18^O; open circles) and carbon (δ^13^C; solid circles) isotope analysis of eight cattle tooth enamel bioapatite following their location from the enamel–root junction (erj).

Results from δ^13^C and δ^18^O measurements in enamel bioapatite are plotted in Fig 4 and summarised in S5 Table in S1 File. The δ^13^C values vary between −13.8‰ and −10.3‰. Using a +14.1‰ ^13^C-enrichment factor between diet and enamel bioapatite [60], these individuals consumed plants with estimated δ^13^C values of –27.9‰ to –24.4‰. The degree of intra-tooth carbon isotopic variation is less than 1‰ in most cases which reflects seasonal changes in the isotopic composition of the animal's diet throughout tooth formation. BOS94 has the highest amplitude (1.4%) and consumed plants with δ^13^C values between −27.9‰ and −25‰ with the lowest value coinciding with a low δ^18^O value (−6.6‰).

The δ^18^O values vary between −6.7‰ and −1.9‰. The amplitude of intra-tooth oxygen isotopic variation is between 0.7‰ (Bos211, Bos132) and 1.8‰ (Bos209). All the sampled individuals yielded a sinusoidal δ^18^O sequence representing at least one complete seasonal cycle, except for Bos170. The periodic cycle measured for this specimen was too short to be modeled; hence we did not use this individual to infer the birth season.

**Fig 5:**
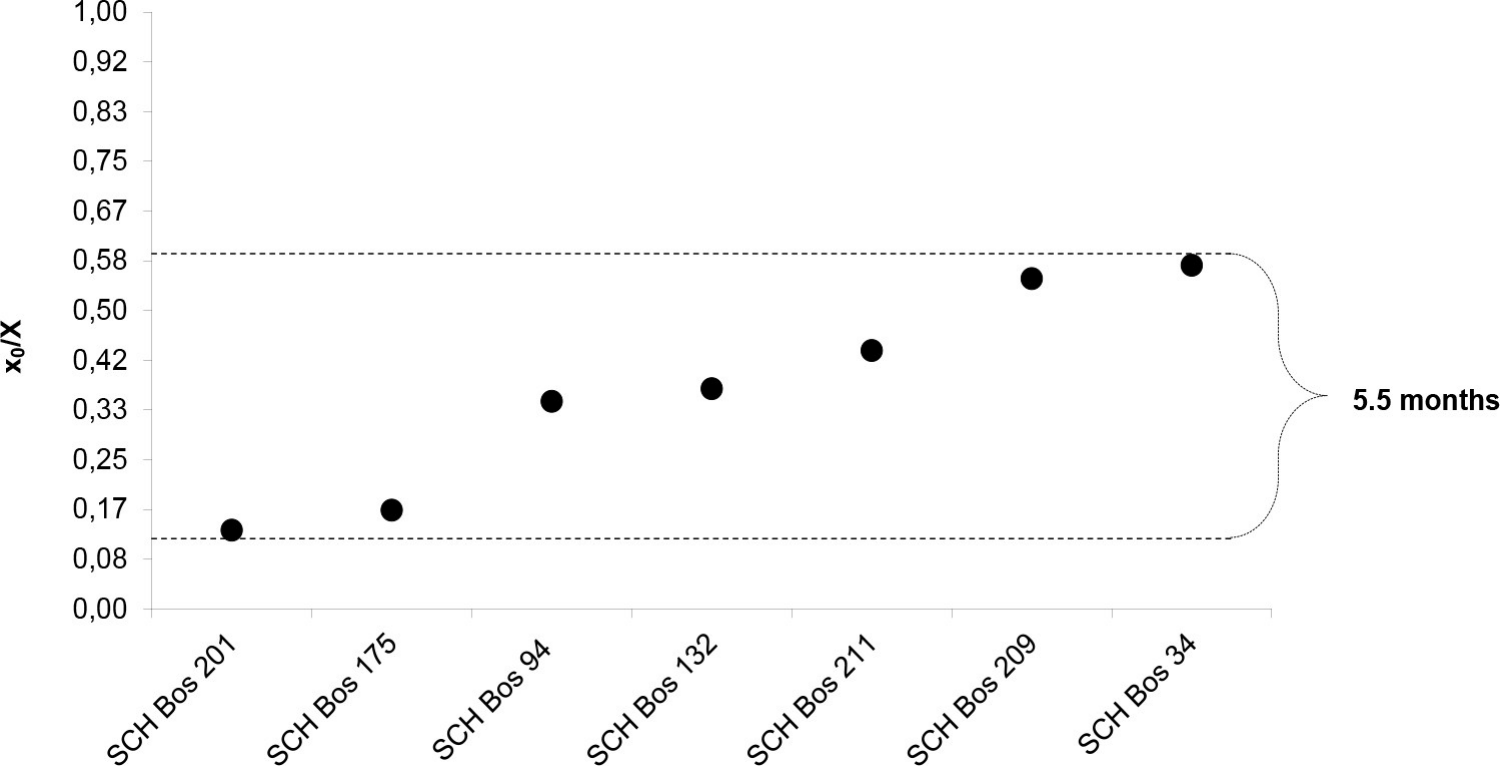
Birth distribution in months of seven cattle from Schipluiden based on the inter-individual variability in the position of the maximum δ^18^O ratio (X_0_) measured in the tooth crown, normalized by the cycle period (X).

Results from the modeling of δ^18^O sequences are shown in Table 1. The length of tooth crown formation over a year (*X*) varies from 34.5 to 54.4 mm, reflecting inter-individual differences in annual tooth growth rate. To remove the influence of this variability, we normalised the locations where the highest δ^18^O value is measured (*x*_*0*_) to the period (crown length formed over a year, *X*), to express the duration of the annual cycle over which all individuals were born (*x*_*0*_/*X*). These values are distributed between 0.13 and 0.58 (range of 0.45) in five specimens, meaning that the analysed cattle were born within 45% of a yearly cycle (12 months), corresponding to a birthing season of approximately five and a half months at Schipluiden (Fig 5). Pearson’s *r*, reflecting the proximity between the modeled data and the measured δ^18^O sequences are between 0.94 and 0.99.

**Table 1:** Results of the calculation of the best fit (method of least squares, applying the Microsoft Excel software Solver function) between the modeled and measured δ^18^O datasets, following the equation in [50].

Specimen	*X* (mm)	*A* (‰)	*xₒ* (mm)	*M* (‰)	*xₒ*/*X*	*r* (Pearson)
SCH BOS94	39.3	0.9	13.7	−5.9	0.35	0.97
SCH BOS175	54.4	1.5	9.0	−3.3	0.17	0.99
SCH BOS201	43.4	1.2	5.7	−4.5	0.13	0.97
SCH BOS34	42.1	1.6	24.3	−4.0	0.58	0.99
SCH BOS132	40.3	0.7	14.9	−5.7	0.37	0.94
SCH BOS211	34.5	0.7	14.9	−5.3	0.43	0.98
SCH BOS209	43.2	1.8	23.9	−4.3	0.55	0.97

X: tooth length formed over a year; A: amplitude; M: mean; x_0_: position in the tooth crown where δ^18^O has the highest value; r: the proximity between the modeled data and the measured δ^18^O values.

### The δ^13^C and δ^15^N measurements in bone collagen

All the samples from mammals yielded good-quality collagen, with N% above 12 and C:N ratios ranging between 3.2 and 3.5 except for one suid which has been excluded from the dataset (Table 2). Among the fish, only three sturgeons (17409, 17410, 17414), contained well-preserved collagen.

**Table 2:** Results from stable carbon (δ^13^C) and nitrogen (δ^15^N) isotope analysis of bone collagen from dog (Canis familiaris), red deer (Cervus elaphus), pig/wild boar (Sus cf. scrofa), cattle (Bos taurus) and Atlantic sturgeon (Acipenser sturio) from Schipluiden.

CIO sample n	Specimens code	Species	Element	%C	%N	C:N	δ^1^ᵌC (‰; IRMS)	δ^1^⁵N (‰; IRMS)
71523	9166	*Canis familiaris*	Femur	36.5	13.4	3.2	−17.9	14.4
71524	2667	*Canis familiaris*	Radius	40.5	14.7	3.2	−16.5	14.9
71525	10681	*Canis familiaris*	Mandible	40.3	14.4	3.3	−18.9	12.3
71526	7564	*Cervus elaphus*	phalanx I	33.3	11.6	3.3	−20.9	11.2
71527	10735	*Cervus elaphus*	Metacarpus	40.3	14.2	3.3	−21.4	5.5
71528	7558	*Cervus elaphus*	Metacarpus	46.6	16.5	3.3	−20.4	8.6
71529	8743	*Cervus elaphus*	Humerus	36.5	12.7	3.3	−20.9	7.0
71530	2321	*Cervus elaphus*	Radius	40.0	13.9	3.4	−21.8	6.8
71531	7660	*Cervus elaphus*	Tibia	48.8	17.2	3.3	−20.5	8.8
71533	10597	*Sus* cf. *scrofa*	Mandible	38.7	13.3	3.4	−21.2	6.5
71534	4542	*Sus scrofa domesticus*	Scapula	42.3	15.1	3.3	−21.5	7.7
71535	7572	*Sus* cf. *scrofa*	Tibia	37.2	13.0	3.3	−21.3	8.0
71536	10737	*Sus* cf. *scrofa*	Tibia	44.4	15.9	3.3	−20.8	8.6
71537	2326	*Sus scrofa domesticus*	Mandible	33.4	11.7	3.3	−22.0	8.5
17362	6419	*Bos taurus*	Metatarsus	39.8	14.2	3.3	−21.5	9.0
17363	6566	*Bos taurus*	Radius	40.5	14.3	3.3	−21.1	9.0
17365	6568	*Bos taurus*	Metatarsus	37.6	13.4	3.3	−20.2	10.7
17368	6080	*Bos taurus*	Metatarsus	41.6	15.0	3.2	−21.4	9.3
17371	7463	*Bos taurus*	Metatarsus	36.9	13.0	3.3	−20.6	9.1
17372	7466	*Bos taurus*	Metatarsus	37.3	13.5	3.2	−21.9	8.6
17373	4590	*Bos taurus*	Metacarpus	40.6	14.7	3.2	−21.1	10.6
17376	8080	*Bos taurus*	Metacarpus	36.1	13.2	3.2	−20.2	9.0
17380	3554	*Bos taurus*	Metatarsus	39.7	14.3	3.2	−21.8	9.0
17383	2136	*Bos taurus*	Metacarpus	35.2	12.8	3.2	−22.5	8.7
17387	2733	*Bos taurus*	Metatarsus	31.5	11.5	3.2	−22.8	7.5
17388	2725	*Bos taurus*	Metacarpus	40.6	14.6	3.3	−20.3	8.8
17390	1681	*Bos taurus*	Metacarpus	36.9	12.5	3.5	−20.7	11.1
17393	1666	*Bos taurus*	Metacarpus	31.8	11.5	3.2	−22.7	9.0
17394	1904	*Bos taurus*	Femur	40.5	14.5	3.3	−20.5	11.6
17397	10163	*Bos taurus*	Metacarpus	41.0	14.6	3.3	−21.2	8.5
17398	09551	*Bos taurus*	Metacarpus	32.7	11.9	3.2	−21.0	8.7
17401	3205	*Bos taurus*	Metatarsus	41.3	15.0	3.2	−22.0	8.5
17405	10372	*Bos taurus*	Metacarpus	39.6	14.2	3.3	−21.6	8.4
17407	9988	*Bos taurus*	Metacarpus	41.3	14.8	3.3	−21.9	4.9
17409	937	*Acipenser sturio*	Operculum	33.9	11.5	3.4	−20.1	11.7
17410	2127	*Acipenser sturio*	Operculum	28.4	9.9	3.3	−14.2	14.8
17414	10418	*Acipenser sturio*	Operculum	29.7	10.2	3.4	−14.8	14.4

**Fig 6:**
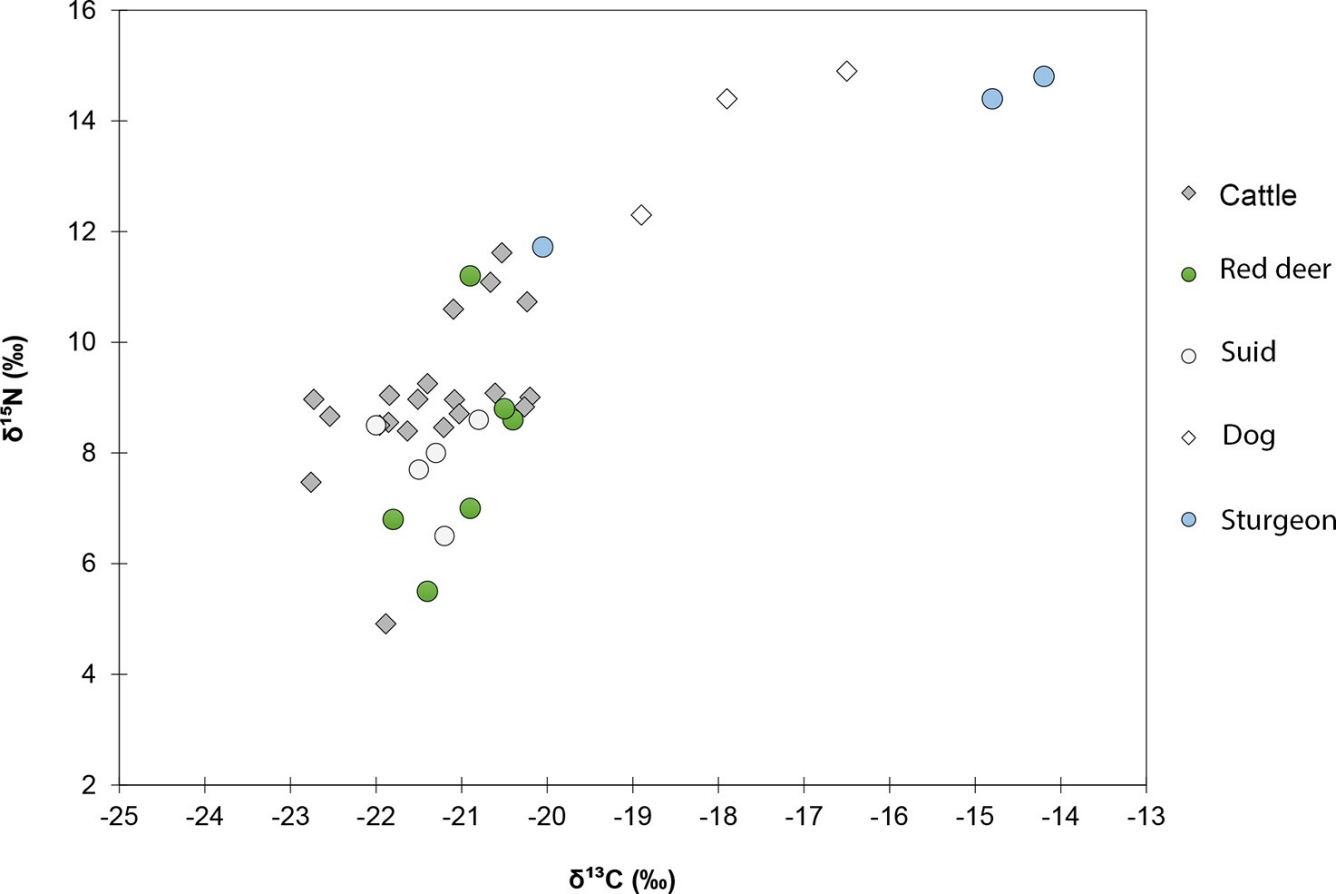
Stable carbon (δ^13^C) and nitrogen (δ^15^N) isotope values of Schipluiden animal bone collagen.

Cattle bone collagen show δ^13^C values between −22.8‰ and −20.2‰ (Fig 6). Bos17383, Bos17387, and Bos17393 stand out with δ^13^C values below −22.5‰. Considering a 5.1‰ ^13^C-enrichment between the protein fraction of diet and collagen [57], Schipluiden cattle had a diet composed of plants with a range of δ^13^C values of –27.9‰ to –25.3‰. Red deer and suids exhibit average δ^13^C values comparable to that of cattle (–21.4‰). Red deer returned δ^13^C values between −21.8‰ and −20.4‰ (–21‰ on average) and suids between −22‰ and −20.8‰ (–21.4‰ on average). In comparison, dogs exhibit average δ^13^C values higher than cattle by 3.6‰, with values ranging between –18.9‰ and –16.5‰. Atlantic sturgeons display a wide range in δ^13^C values for anadromous (migratory) fish (−20.1‰ to −14.2‰).

The δ^15^N values measured in cattle bone collagen are exceptionally high, ranging from 7.5‰ to 11.6‰, with the majority clustering between 8.5‰ and 9.2‰. BOS17407 stands out with the lowest δ^15^N value of 4.9‰. Red deer and suids display average δ^15^N values slightly lower than that of cattle (9‰) by _~_1‰, representing a clear overlap in the range of measured δ^15^N values for herbivores and omnivores. Red deer δ^15^N values range between 5.5‰ and 11.2‰ (8‰ on average). Suids have a range of δ^15^N values between 6.5‰ and 9.5‰ (8.1‰ on average). δ^15^N values as high as 12.3‰ to 14.9‰ were measured in dog bone collagen. Sturgeon display the highest δ^15^N values within the dataset, ranging between 11.7‰ and 14.8‰ (14.1‰ on average). The δ^15^N values measured in the faunal remains align with expected trophic levels, with fish occupying the highest trophic level, followed by dogs, as carnivores. Omnivores and herbivores show relatively lower δ^15^N values, however, their elevated δ^15^N values to what would be expected is notable.

## Discussion

The established role of cattle husbandry in Schipluiden, as the earliest known Neolithic settlement in the Lower Rhine-Meuse area, is demonstrated by the dominance of morphologically domestic cattle in the archaeological assemblage [30]. The different aspects of cattle husbandry, in terms of slaughtering pattern, calving season, and foddering management are of great importance for understanding the early human-cattle relationships is the Dutch wetlands in the mid-4^th^ millennium BC.

Age-at-death analysis suggests sophisticated cattle husbandry in Schipluiden in which animals were managed for meat and milk production. Meat exploitation appears to be one of the central foci of Schipluiden cattle husbandry, through the slaughtering of a large proportion of animals at their peak weight. The limited number of morphologically wild cattle bones may represent aurochs occasionally hunted as a supplemental meat to domestic cattle. Evidence for a high number of adult females in the assemblage based on biometric analysis of metacarpals, together with slaughtering animals in the age class of 6‒8 years indicates milk production. This inference is supported by the abundance of slaughtering “post-lactation” calves between 6–15 months, implicating that a high number of calves were kept alive to ensure milk let-down. Based on the fusion data, among 321 cattle long bones studied previously [31], 124 are reported to be unfused. Given that all cattle long bones are fused by roughly 48 months [77], a large number of cattle appear to have been culled before reaching maturity. Culling these individuals at the end of lactation would also reduce the herd at the beginning of the winter, mitigating the scarcity of pasture and fodder and increasing the meat return [79].

The high frequency of infants in age class 0–1 month at Schipluiden may be due to natural mortality, which mainly occurs within the first month of life during winter calving, primarily due to exposure [80]. The lack of older cattle at Schipluiden and the minimal osteological pathologies reported [31] imply that cattle were not used for traction. Similar cattle mortality profiles are reported from sites dating to the 6^th^ to 4^th^ millennium BC in central and northern Europe [79,81,82]. Post-lactation slaughtering of calves associated with milk production has been demonstrated at Neolithic sites elsewhere in Europe [76,82] strongly indicating the use of cattle meat and milk from the onset of the Neolithic in these regions.

The length of cattle birth season at Schipluiden, based on stable oxygen isotopes was around five and a half months, corroborating the inference that milk production was an important purpose for cattle farming at Schipluiden. This result makes the calving season in Schipluiden much longer than that of modern primitive cattle breeds, for example in Scotland, spanning over 2–3 months [47]. Cattle birth seasonality displays variability across Neolithic Europe. At the Linearbandkeramik (LBK) site of Chotĕbudice (late 6^th^ millennium BC, Bohemia, Czech Republic), calving season was short (2–3 months) [68], whereas, at Neolithic Bercy (4^th^ Millennium BC, Paris Basin, France) calving season is reported to be about six months [15]. Variations in the seasonality of birth have also been observed among the Neolithic cattle populations in Scotland [46]. Environmental constraints, seasonal scarcity of fodder, as well as biological and cultural factors, can play an important role in creating these variations [46]. To what extent the prolonged birthing season at Schipluiden was related to the physiology of the animals, the environment, and the cultural preferences of the herders cannot be established here. However, the mixed mortality profile of cattle at the site suggests that the farmers might have modulated the mating season for a longer availability of milk as has been proposed at Bercy [15]. The lactation span of modern European primitive cows is reported to be approximately 6–7 months [83]. The extension of the calving season at Schipluiden prolonged the availability of milk to be processed into dairy products, which would have provided major sources of protein for non-lactase persistent populations [84,85] during winter or in case of crop failure. Considering the temporal breadth covered by the samples, of around 200 years, and the small sample size (n = 7) this interpretation should be approached with caution. Pearson’s *r* measurements, however, suggest the high accuracy of the modeled data based on the measured δ^18^O sequence.

Milk processing can also be evidenced by the organic residue analysis of ceramics [2,86]. So far, the organic residue analysis on the ceramic vessels from Schipluiden involved the analysis of charred food remains using direct temperature-resolved-mass spectrometry (DT-MS) and showed freshwater fish components [87]. Milk indicators are detected by lipid extraction and gas chromatography combustion-isotope ratio mass spectrometry (GC-C-IRMS) [88]. By using this method on Neolithic vessels from England [89], Denmark [90], and LBK sites in northern Europe [91], the beginning of dairy production in temperate Europe has been pushed back to the 6^th^ millennium BC. The recent discovery of milk protein in human dental calculus in Neolithic Britain has also provided direct evidence for the consumption of dairy products [92]. While we recommend further study of the ceramic vessels and dental calculus from Schipluiden, we note that the absence of milk residues in ceramic potsherds does not preclude the use of non-ceramic, perishable containers for processing milk.

### Management of cattle diet

Schipluiden cattle appear to have pastured in an isotopically similar environment to red deer and suids, possibly at the beach plain where C_3_ plants were abundant. Four cattle individuals (Bos94, Bos17383, Bos17387, and Bos17393), had δ^13^C values as low as –27.9‰ for their diet. This is lower than the δ^13^C value of –27.5‰ as the endpoint for grazing on pre-industrial C_3_ plants in open areas [59]. Similar estimated dietary δ^13^C values (–27.2‰ to –26.4‰) have been recorded from the Late Mesolithic aurochs in Balkweg (5680–5520 BC) and Jardinga (5570–5370 BC) in the northern Netherlands [93,94]. These values have been interpreted as the result of aurochs grazing in dense woodlands or water-rich environments. Pollen analysis reconstructed Schipluiden as a treeless dune [35]. Tree species, including alder (*Alnus* sp.*)*, willow (*Salix* sp.), apple (Pomoideae), sloe (*Prunus spinose*), juniper (*Juniperus communis*), and ash (*Fraxinus* sp.) have been recovered at the site in the form of branches and twigs [95]. Cattle coprolites predominantly contain pollen from grasses (Poaceae) but also from tree species including pine (*Pinus*), oak (*Quercus*), lime (*Tilia*), and hazel (*Corylus*) albeit in smaller proportions [35]. This pollen represents both the diet of the animal and its surrounding environment. We might, therefore, suggest that farmers occasionally provided some cattle with collected fodder from woodlands to maintain the herd during cold seasons. One individual (BOS94) has low δ^13^C values which coincide with low δ^18^O values, suggesting leaf foddering occurred during the cold season. The absence of an observed canopy effect in the δ^13^C values of red deer and suids makes foddering some cattle with leafy hay a more plausible scenario than animals grazing in waterlogged areas.

This picture makes Schipluiden cattle husbandry comparable to some LBK sites in central Europe (e.g. [69,96,97]), where bone collagen δ^13^C values suggest that cattle herding predominantly took place in open areas and /or open forests, with occasional use of dense forest resources. Low δ^13^C values, which reflect the use of forest resources, have been previously observed elsewhere in temperate Europe including the LBK sites of Bischoffsheim (Alsace, France), and Vaihingen (southwestern Germany [64,98]). At the Neolithic site of Weier (Switzerland), twigs have been identified in cattle dung, reaffirming the practice of leaf-foddering [99,100]. Collection of leafy hays may have been practiced because of their high forage quality, which meets the phosphorus requirement of lactating cows and reinforces oxytocin release during milking [101].

The δ^15^N values measured in cattle and red deer bone collagen in our dataset are exceptionally high in comparison with contemporary populations of cattle [98]. Factors that can cause substantial ^15^N enrichment include suckling [102,103], stress and starvation [104], soil manuring [63,105,106], and soil salinity [71,107]. Through the consumption of milk protein, suckling increases the δ^15^N values of infants by one trophic level (2‰–3‰) in comparison to their mothers [108]. In our dataset suckling cannot be an issue as we sampled subadult or adult individuals. The highest δ^15^N value (11.6‰) was measured in a cattle femur with fused distal epiphysis (i.e. an individual older than 4 years). Another cause of elevated stable nitrogen isotopic values may be stress or starvation [104], which may be evident from osteological pathologies. Only one excessive bone growth on the cavity (acetabulum) of a bovine pelvis is observed [31], which is not definitive evidence for dietary stress.

Long-term incorporation of manure into croplands to increase the fertility of the soil can increase the ^15^N value of plants by 4‰ or higher, depending on the intensity of manuring. This process in return elevates the δ^15^N values in humans and animals who consume the plants growing in this environment [63,105,109]. Long-term use of winter pasture is reported to have the same influence on the nitrogen level in the dentine collagen of molars in modern sheep flocks in Mongolia [110]. We cannot establish the degree to which manuring may have impacted the soil around Schipluiden without further analysis of the cereals. The saline environment of the dune, as shown by palaeoenvironmental studies [35,111], is probably the most significant factor elevating δ^15^N values of cattle grazing in this environment. The positive correlation between soil salinity and the ^15^N ratio of coastal and salt marsh plants is well documented [71,112]. Overall, these findings suggest that cattle were reared in the salt marsh and the beach plain near the dune. In this environment, the sea spray effect [112] shifted the isotopic signals of cattle and all other terrestrial fauna living in coastal regions towards marine values.

### Reanalysis of the human palaeodiet at the site

**Fig 7:**
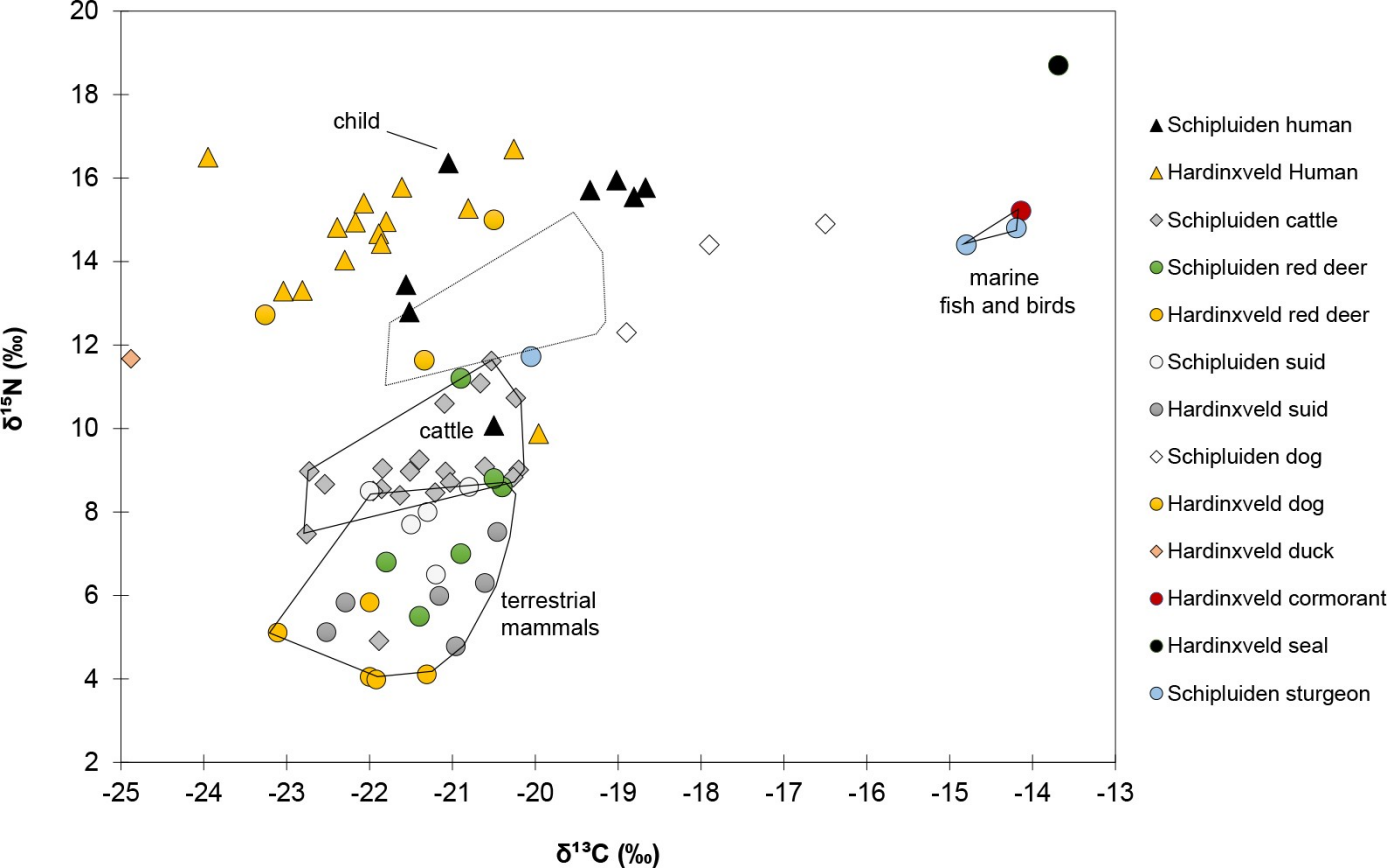
δ^13^C and δ^15^N values of bone collagen from Schipluiden fauna (this study), humans (n = 8), De Bruin, and Polderweg (Hardinxvel humans n = 14, fauna n = 14)). Data from [24,25].

With this study, we have extended the faunal stable isotope baseline for Schipluiden, which enables us to revisit the previous inferences on human palaeodiet at the site. Interesting patterns arise when we compare our faunal dataset with previously reported human and faunal isotopic data from the Mesolithic sites of De Bruin and Polderweg and those from Schipluiden [24,25]. We merged the stable isotope values at the Mesolithic sites as they are contemporaneous and are in close geographic proximity (Hardinxveld), excluding a total of 14 reported measurements in which their collagen quality and C:N ratios were outside of the generally accepted ranges of 2.9 to 3.6 [113]. The remaining 14 human bones from De Bruin and Polderweg exhibit δ^13^C values between –24.0‰ and –20.0‰ and δ^15^N values between 9.9‰ and 16.7‰ (Fig 7). These depleted δ^13^C values and enriched δ^15^N values can be explained by the consumption of riverine fish and waterfowl as a prominent source of protein. Marine species, as represented in the dataset by one seal (*Phoca* sp.) and one cormorant (*Phalacrocorax* sp.) exhibit high δ^13^C (–14.1‰ and –13.7‰ respectively) and δ^15^N (15.2‰ and 18.7‰ respectively) which supports our interpretation that they did not play an important role in the Mesolithic diet. Evidence for this interpretation derives from the location of the sites along a river and far away from the contemporaneous seashore and the abundant zooarchaeological evidence for freshwater species [33,34]. The riverine environment of the site might have also contributed to δ^13^C depletion in these individuals [69].

In comparison, seven human adults from Schipluiden exhibit δ^13^C values between –21.6‰ and –18.7‰ (–19.9‰ on average), scattering between the endpoints for pure terrestrial and marine diet [72]. The δ^15^N values are as high as 10.1‰ to 16.0‰ (14.2‰ on average). Excluding the data from a 2-year-old child that exhibits the highest δ^15^N (16.4‰) in the dataset, possibly as a result of suckling [103], the δ^15^N values of the human bones from Schipluiden are unusually higher than other Neolithic populations documented in temperate Europe [8,114–116]. The first study that tackled human subsistence at Schipluiden, interpreted these values to indicate marine consumption [24,25]. In this study, we show that the terrestrial mammals at the site also exhibit enriched δ^15^N values, compelling a reconsideration of the original interpretation. Schipluiden human remains exhibit on average δ^15^N values that are 5.2‰ higher than those from cattle remains. In part, we can suggest that the elevated δ^15^N values measured in human remains can be explained by the consumption of terrestrial mammals, mainly cattle with elevated δ^15^N values. Human bone collagen from Neolithic Orkney (4^th^ millennium BC) exhibit similar δ^13^C values which have been interpreted as a result of consumption of products from animals grazing on marine seaweed which in turn would increase their δ^13^C values substantially [117]. The δ^13^C values of Schipluiden cattle bone collagen and tooth bioapatite are in the expected range of terrestrial C_3_ plants and does not suggest any seaweed consumption. Overall, in contrast to the previous study, we suggest that Schipluiden had a mixed terrestrial and marine protein diet but that the marine protein input was relatively small in comparison to terrestrial resources. The high δ^15^N values observed at Schipluiden resulting not only from consuming marine resources but also from substantial consumption of terrestrial plants and animals, such as cattle, from a ^15^N-enriched environment.

This interpretation of how human palaeodiet at Schipluiden was constructed is consistent with previous and current zooarchaeological results from Schipluiden, which both indicate a substantial consumption of cattle protein since the onset of farming at the site [31]. This picture challenges the proposed “gradual adoption of the Neolithic way of life” based on the continuity of an aquatic-based diet in the Neolithic Dutch Wetland [22,24,118]. Our data reveal a rapid departure from the Mesolithic way of life towards an economy in which cattle gained social and economic importance with the onset of farming in the region in the mid-4^th^ millennium BC.

## Conclusions

Schipluiden, the earliest known year-round settlement at the Rhine-Meuse delta, is a key site for investigating the Neolithic way of life in Northwest Europe. Using zooarchaeological remains we show that cattle husbandry was an integral component of Schipluiden subsistence. Cattle meat and milk production were achieved by culling male cattle near or at their optimum weight while probably maintaining female cows until older ages. Post-lactation slaughtering of calves guaranteed lactating cow’s milk let-down. Farmers could control the birthing season of cattle through fodder provision, extending the availability of milk for human consumption during food scarcity or crop failure. Cattle were mainly kept in the vicinity of the site where C_3_ plants were abundant with leaf-fodder providing a limited contribution to the diet of some cattle. The major investment in maintaining this animal would have also strengthened its social importance for the community, as is evidenced by the special attention paid to cattle body disposition.

Using the faunal isotopic data, we show that the high ^15^N in human bone collagen is more likely to signal the consumption of products deriving from cattle which grazed on ^15^N-enriched salt marsh plants around the site than a marine-based diet. This contradicts the previous interpretation of the subsistence at Schipluiden being a continuation of marine food consumption long after the introduction of farming. Results presented in this study suggest that human diet in the mid-4^th^ millennium BC Rhine-Meuse area was primarily based on products from domesticates, especially cattle, with some input from the available wild terrestrial and aquatic resources. This changes the current narratives of Neolithic lifeways in the Rhine-Meuse Delta and urges further interdisciplinary research for understanding the nature and role of early animal husbandry in this region.

## Supporting information

S1 File (DOCX)
