# Supplementary material for: Specialized cattle farming in the Neolithic Rhine-Meuse Delta: Results from zooarchaeological and stable isotope (δ18O, δ13C, δ15N) analyses
Source: PLoS One. 2020 Oct 21;15(10):e0240464. doi: 10.1371/journal.pone.0240464 (PMC7577484; doi:10.1371/journal.pone.0240464)
Supplement: S1 File — (DOCX) [file pone.0240464.s001.docx]

|  | **M1** | | **M2** | |  |  |  |  |  |
| --- | --- | --- | --- | --- | --- | --- | --- | --- | --- |
| **Mandible code** | **WA (mm)** | **CervL (mm)** | **WA (mm)** | **CervL (mm)** |  | **Tooth code** | **WA (mm)** | **CervL (mm)** | **Identified as** |
| 1 | 16.8 | 24.7 | 16.7 | 26.8 |  | 14 | 14.9 | 23.3 | M1 |
| 6 | 15.6 | 21.6 | 17.2 | 24.3 |  | 40 | 14.8 | 21.2 | M1 |
| 8 | 15.7 | 24.2 |  |  |  | 54 | 15.2 | 24.3 | M1 |
| 9 | 15.4 | 21.9 | 16.4 | 25 |  | 101 | 15.7 | 23.5 | M1 |
| 15 | 16.3 | 21.6 | 17.6 | 24.9 |  | 106 | 15.7 | 22.9 | M1 |
| 28 | 17.2 | 24.9 |  |  |  | 122 | 14.6 | 23.4 | M1 |
| 31 | 15.6 | 21.7 | 15.9 | 25 |  | 135 | 15.5 | 23 | M1 |
| 33 | 15.5 | 22.7 |  |  |  | 149 | 14.3 | 21.7 | M1 |
| 55 | 15.8 | 23.5 |  |  |  | 150 | 15 | 23 | M1 |
| 77 | 15.1 | 22.3 |  |  |  | 178 | 15.2 | 22.8 | M1 |
| 91 | 16.1 | 22.7 | 17 | 25.3 |  | 192 | 14.3 | 21.9 | M1 |
| 95 | 16.8 | 23.7 | 17.3 | 26.4 |  | 195 | 15.7 | 23.5 | M1 |
| 96 | 15.9 | 21.7 | 16 | 24.5 |  | 199 | 16.1 | 22.3 | M1 |
| 107 | 15.9 | 23.6 | 16.2 | 25.9 |  | 204 | 16.6 | 23 | M1 |
| 108 | 15.6 | 21.9 |  |  |  | 207 | 15.7 | 22.4 | M1 |
| 134 | 15.4 | 22.3 |  |  |  | 47 | 15.9 | 27.2 | M2 |
| 136 | 14.3 | 22.4 |  |  |  | 83 | 16 | 26.3 | M2 |
| 143 | 16.2 | 23 |  |  |  | 104 | 16.9 | 26.1 | M2 |
| 147 | 15.1 | 21 | 15.7 | 24.5 |  | 112 | 17.1 | 26.8 | M2 |
| 151 | 15.9 | 23.7 |  |  |  | 123 | 16.5 | 27.6 | M2 |
| 163 | 15.7 | 23 |  |  |  | 124 | 16 | 25 | M2 |
| 167 | 15.2 | 21.9 | 15.4 | 24.5 |  | 130 | 16.1 | 24.5 | M2 |
| 170 | 15.7 | 24 | 16.9 | 25.8 |  | 140 | 16.4 | 24.4 | M2 |
| 171 | 16.3 | 24.6 |  |  |  | 173 | 17.4 | 27.9 | M2 |
| 174 | 15.4 | 23.2 |  |  |  | 176 | 15.8 | 25.8 | M2 |
| 191 | 15.1 | 21.2 | 15.7 | 24.5 |  | 182 | 16.7 | 25.1 | M2 |
| 201 | 17 | 24.5 | 17.7 | 26.7 |  | 185 | 16.2 | 24 | M2 |
| 203 |  |  |  |  |  | 186 | 15 | 25.9 | M2 |
|  |  |  |  |  |  | 188 | 16.3 | 27 | M2 |
|  |  |  |  |  |  | 200 | 16.1 | 26.1 | M2 |

S1 Table. Left: Cervical length (CervL) relative to the width of anterior (WA) in intact M1 and M2, following Beasley et al. (74) as reference for the identification of loose molars (right).

*
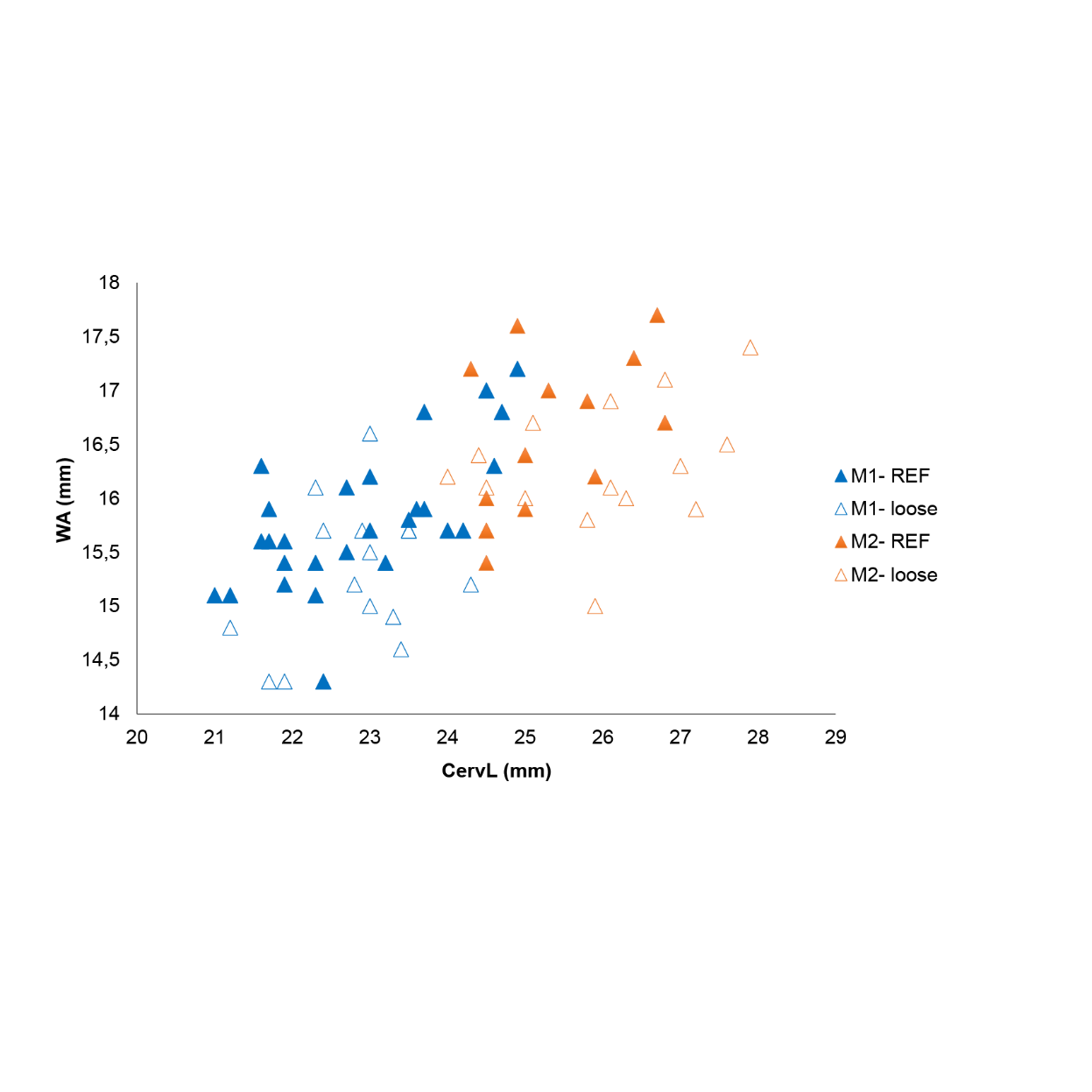
*

*S1 Fig. Distinguishing between loose first (M1) and second (M2) mandibular molars of domestic cattle found at Schipluiden by comparing the length of the cervical (CervL) to the width of the anterior (WA) (measurements after (74)). Measurements on embedded M1 and M2 from complete mandibles in the assemblage (represented by closed triangles) are shown for reference.*

|  |  | **Grant's age classes** | | | | |  |  |  | **Grant's age class** | | | | |  |
| --- | --- | --- | --- | --- | --- | --- | --- | --- | --- | --- | --- | --- | --- | --- | --- |
| **find number** | **Side** | **dp4** | **P4** | **M1** | **M2** | **M3** | **Legge's Stage** | **find number** | **Side** | **dp4** | **P4** | **M1** | **M2** | **M3** | **Legge's Stage** |
| 9510 | R | a |  |  |  |  | 1 | 1071 | L |  |  | d |  |  | 4 |
| 10271 | R | a |  |  |  |  | 1 | 2820 | R |  |  | c |  |  | 4 |
| 9523 | L | b |  |  |  |  | 1 | 7099 | L |  |  | f | a |  | 4 |
| 6558 | L | f |  |  |  |  | 2 | 9515 | L | j |  | d |  |  | 4 |
| 6741 | L |  |  | a |  |  | 3 | 4504 | R |  |  | d |  |  | 4 |
| 8625 | R | e |  |  |  |  | 3 | 4505 | R | j |  |  |  |  | 4 |
| 3747 | L |  |  | b |  |  | 3 | 1370 | R | f |  |  |  |  | 4 |
| 2419 | L |  |  | a |  |  | 3 | 9181 | R | f |  |  |  |  | 4 |
| 3161 | R |  |  | a |  |  | 3 | 2733 | R | f |  |  |  |  | 4 |
| 7816 | L | j |  | e | a |  | 4 | 9931 | R | j |  | g | b |  | 5 |
| 7476 | R |  |  | c |  |  | 4 | 7813 | R | k |  |  |  |  | 5 |
| 7817 | R | j |  | d |  |  | 4 | 4263 | R | j |  | g | c |  | 5 |
| 7281 | R | j |  | d | C |  | 4 | 7494 | R | j |  | g | b | V | 5 |
| 1689 | L |  |  | b |  |  | 4 | 3554 | L | j |  | g |  |  | 5 |
| 1677 | L |  |  | f |  |  | 4 | 1452 | R | k |  |  |  |  | 5 |
| 9527 | L | j |  | b |  |  | 4 | 1618 | L |  |  |  | e |  | 5 |
| 9512 | L | j |  |  |  |  | 4 | 1505 | L |  |  |  | b |  | 5 |
| 9264 | R | f |  | b |  |  | 4 | 9520 | L |  |  | h |  |  | 5 |
| 8540 | R |  |  | e |  |  | 4 | 9102 | L |  |  | e |  |  | 5 |
| 10630 | L | j |  |  |  |  | 4 | 8505 | L |  |  |  | e |  | 5 |
| 2738 | R |  |  | d |  |  | 4 | 2183 | R | j |  |  |  |  | 5 |
| 6073 | R | j |  | d | C |  | 4 | 7010 | L | j |  | e | b |  | 5 |
| 10046 | L |  |  | b |  |  | 4 | 5127 | R | k |  | g | b |  | 5 |
| 9897 | L | j |  |  |  |  | 4 | 10118 | R |  |  | g | e |  | 5 |
| 8069 | L | j |  | e | E |  | 4 | 3392 | L |  |  | f |  |  | 5 |
| 8659 | L | j |  | d |  |  | 4 | 4489 | L |  |  |  | b |  | 5 |
| 9509 | R | f |  |  |  |  | 4 | 8334 | R |  |  |  | c |  | 5 |
| 4593 | L | j |  | e |  |  | 4 | 10238 | R |  |  | g |  |  | 5 |
| 10598 | L | j |  | c |  |  | 4 | 10456 | R |  |  | g |  |  | 5 |
| 10329 | R |  |  | f |  |  | 4 | 6348 | R |  |  |  | b |  | 5 |
| 10456 | L | j |  |  |  |  | 4 | 9411 | R |  |  | h |  |  | 5 |
| 5965 | R | j |  | f |  |  | 4 | 5610 | L | k |  |  |  |  | 5 |
| 5682 | L |  |  | e |  |  | 4 | 3386 | R |  |  |  |  |  | 5 |
| 5589 | L |  |  | f |  |  | 4 | 3382 | R |  |  | g | d |  | 5 |
| 1289 | R | j |  | d |  |  | 4 | 4982 | R |  |  |  |  | c | 5 |
| 10324 | R |  |  | a |  |  | 4 | 9937 | R |  |  |  | c |  | 5 |
| 328 | R | j |  |  |  |  | 4 | 3839 | R |  |  | g |  |  | 5 |
| 5242 | L | j |  | d |  |  | 4 | 7887 | L |  |  |  |  | a | 5 |
| 9470 | R | j |  | d | U |  | 4 | 8851 | R |  |  | h |  |  | 5 |
| 3777 | R | j |  |  |  |  | 4 | 8433 | R |  |  |  | c |  | 5 |
| 3777 | R |  |  | a |  |  | 4 | 2344 | L |  |  | h | c |  | 5 |
| 4351 | L | j |  |  |  |  | 4 | 2818 | R |  |  | g |  |  | 5 |
| 5193 | R |  |  | e |  |  | 4 | 2818 | L |  |  |  | c |  | 5 |
| 8753 | R |  |  |  | a |  | 4 | 3128 | R |  |  | h |  |  | 5 |

|  |  | **Grant's age class** | | | | |  |
| --- | --- | --- | --- | --- | --- | --- | --- |
| **find number** | **Side** | **dp4** | **P4** | **M1** | **M2** | **M3** | **Legge's Stage** |
| 3108 | R |  |  |  | b |  | 5 |
| 4088 | R |  |  | g | d |  | 5 |
| 1820 | R | k |  | g | d |  | 5 |
| 9510 | L |  |  | g |  |  | 5 |
| 2100 | R | k |  |  |  |  | 5 |
| 10119 | L |  | V | k | f | d | 6 |
| 1477 | R |  |  |  |  | c | 6 |
| 8592 | L |  |  |  |  | f | 6 |
| 9808 | R |  |  |  | g |  | 6 |
| 8628 | L |  |  |  | g |  | 6 |
| 8658 | R |  |  |  |  | f | 6 |
| 4756 | L |  |  |  |  | d | 6 |
| 3391 | L |  |  |  |  | c | 6 |
| 4468 | L |  |  |  | g |  | 6 |
| 10596 | L |  |  |  |  | d | 6 |
| 5728 | R |  | f | j | g | cd | 6 |
| 10525 | R |  |  |  |  | b | 6 |
| 6512 | L |  |  | j | g | b | 6 |
| 6508 | R | k | E | j | g |  | 6 |
| 7227 | R |  |  |  |  | b | 6 |
| 4538 | L |  |  | j | g | d | 6 |
| 2760 | R |  | e | k | g |  | 6 |
| 1616 | L |  |  |  | g |  | 6 |
| 7204 | R |  | f | k | g |  | 7 |
| 1496 | L |  |  | j |  |  | 7 |
| 9521 | L |  | b |  |  |  | 7 |
| 6072 | L |  | d | k | j | f | 7 |
| 6072 | L |  | f | k | j | g | 7 |
| 7704 | R |  |  |  |  | g | 7 |
| 5885 | R |  | f |  |  |  | 7 |
| 6579 | R |  | f | k | h | h | 7 |
| 9777 | R |  |  | k |  |  | 7 |
| 5110 | R |  |  |  | j |  | 7 |
| 4655 | L |  | d |  |  |  | 7 |
| 3286 | R |  | e | j | h | f | 7 |
| 7678 | R |  |  | k |  |  | 7 |
| 8712 | L |  |  |  |  | g | 7 |
| 7486 | R |  |  | l | k | j | 8 |
| 7454 | L |  |  | l | k | k | 8 |
| 6780 | R |  |  |  |  | k | 8 |
| 2136 | L |  |  | l | k | k | 8 |
| 9406 | L |  |  |  |  | k | 8 |
| 10271 | R |  |  |  | k |  | 8 |
| 635 | L |  |  |  |  | j | 8 |
| 373 | L |  |  | l |  |  | 8 |

S2 Table. Cattle mandibular tooth eruption and wear stages in Schipluiden following Grant (75) and Legge (46).

| **Tooth code** | **WA (mm)** | **CervL (mm)** | **Note** |
| --- | --- | --- | --- |
| 15 | 15.5 | 36.2 |  |
| 91 | 17.4 | 38.7 |  |
| 113 | 16.1 | 36.5 |  |
| 120 | 16.6 | 38.3 |  |
| 121 | 17.5 | 38.4 |  |
| 147 | 15.1 | 36.9 |  |
| 153 | 16.2 | 37.2 |  |
| 168 | 15.2 | 36.9 |  |
| 190 | 15.9 | 37.1 |  |
| 205 | 16 | 34.9 |  |
| 34 | 16.4 | 40 | Sampled for SI |
| 94 | 16.09 | 36.2 | Sampled for SI |
| 132 | 15.8 | 36.8 | Sampled for SI |
| 170 | 16.8 | 39.4 | Sampled for SI |
| 175 | 16.7 | 39.8 | Sampled for SI |
| 201 | 17.5 | 41.8 | Sampled for SI |
| 209 | 15.7 | 39.2 | Sampled for SI |
| 211 | 14.1 | 34 | Sampled for SI |

S3 Table. Cervical length and width of anterior of cattle lower third molars in Schipluiden used for size assessment and sample selection for stable carbon and oxygen isotope analysis. SI = stable isotopes. Measurements following Beasley et al. (74)


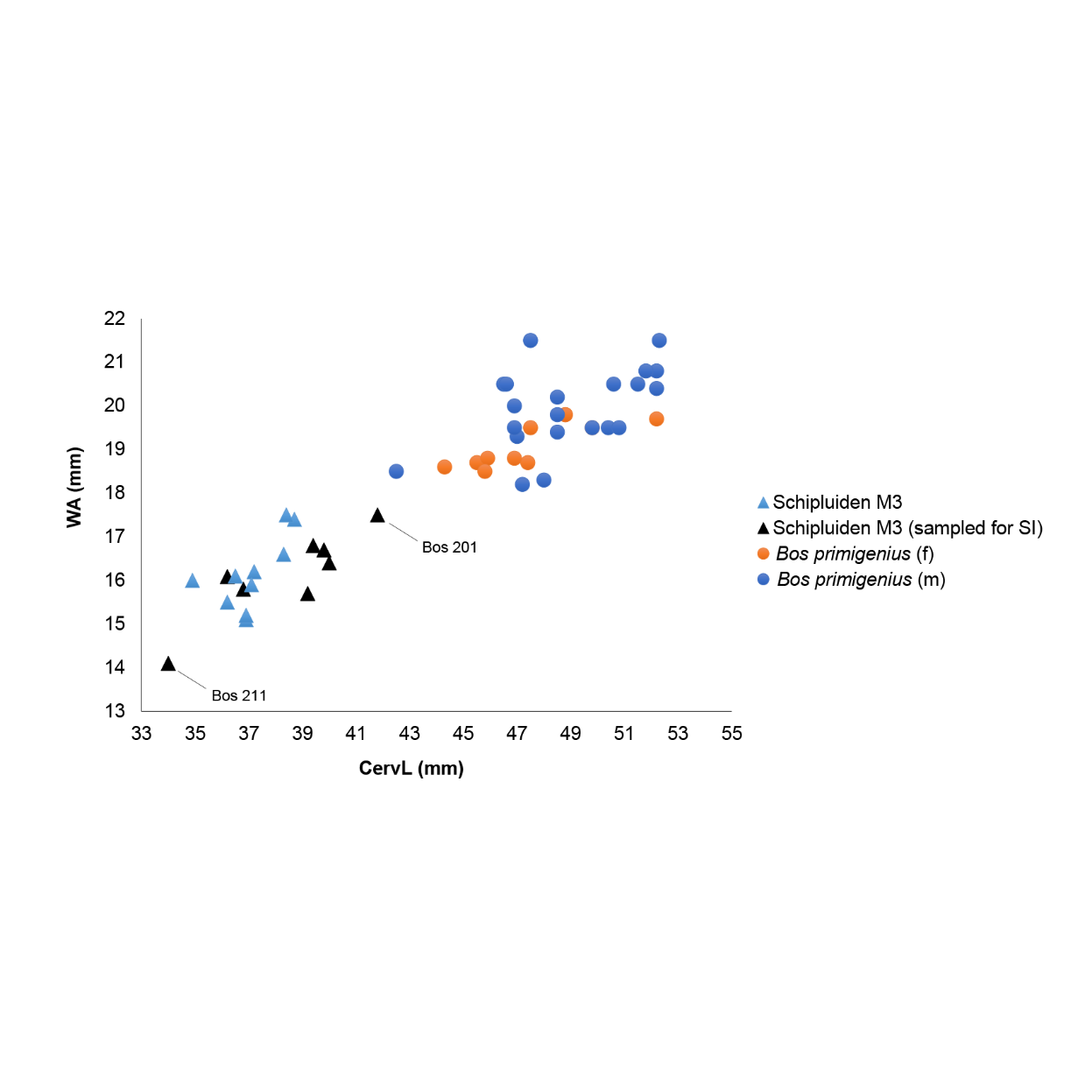


*S2 Fig. Cervical length and width of anterior (following Beasley et al. (74)) of 18 lower third molars from Schipluiden, compared to those from 32 aurochs lower third molar measurements from Denmark (79). SI = stable isotopes; f = female; m= male.*

| **Tooth code** | **find number** | **Dent** | **Age** | **No of sequences** | **Photo** |
| --- | --- | --- | --- | --- | --- |
| BOS170 | 5728 | M3- R | 26-36 m | 17- anterior lobe | 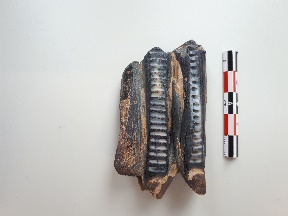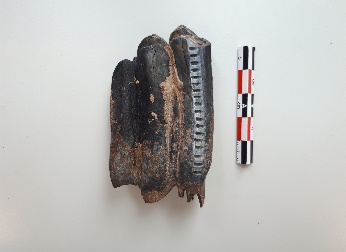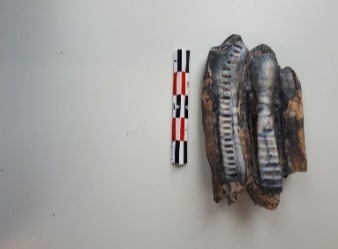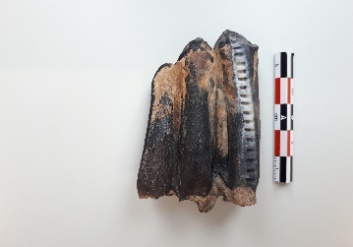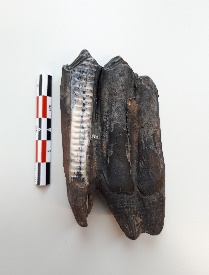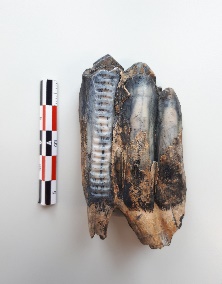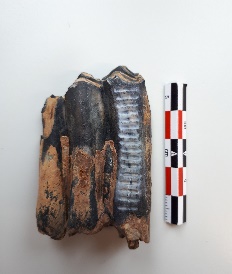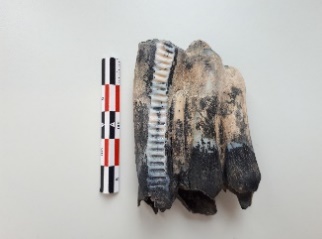   \|  \| \| --- \| |
| BOS34 | 1477 | M3- R | 26-36 m | 18- anterior lobe |  |
| BOS209 | 4538 | M3- L | 26-36 m | 22- anterior lobe |  |
| BOS175 | 10525 | M3- R | 26-36 m | 17- anterior lobe |  |
| BOS94 | 6072 | M3- L | 3-6 y | 20- anterior lobe |  |
| BOS132 | 10596 | M3- L | 26-36 m | 19- anterior lobe |  |
| BOS211 | 2760 | M3- R | 26-36 m | 16- anterior lobe |  |
| BOS201 | 6512 | M3- L | 26-36 m | 22- anterior lobe |  |

*S4 Table. Schipluiden cattle third molars sampled for stable oxygen (δ^18^O) and carbon (δ^13^C) isotope analyses of enamel bioapatite. Ageing following Legge (46).*

**S1 Text. Enamel bioapatite preparation and stable oxygen and carbon isotope analysis**

The sampling was undertaken at the Centre for Isotope Research (CIO), University of Groningen, following the protocol in Balasse (59). Stable isotope analyses were conducted in the Service de Spectrométrie de Masse Isotopique du Muséum national d'Histoire naturelle (SSMIM) in Paris. Samples were treated in 0.1 M acetic acid for four hours and rinsed five times with demineralized water. Bioapatite samples weighing between 600 to 650 ϻg was reacted for 240 s with 100% phosphoric acid at 70 ̊C in individual vessels in an automated cryogenic distillation system (Kiel IV device), interfaced with a Delta V Advantage Isotope Ratio Mass Spectrometer (IRMS). Throughout the analysis, sample results with an interval of ten were checked with laboratory carbonate standards (MarbreLM) to assure the accuracy of the measurements. The corrections applied to the results were between −0.045‰ and +0.16‰.

**S6 Text. Bone collagen preparation and stable carbon and nitrogen isotope analysis**

 Collagen was extracted from 3–5 g of bone tissue and prepared following the acid-base-acid (ABA) treatment described in Kuitems et al. (1), an improved version of the method described in Longin (2). To remove the inorganic components, the bone tissues were soaked in 4% (v/v) hydrochloric acid (HCl) overnight (in both acid steps). In the next step, 1% (v/v) of NaOH was used for the base step. The dissolved collagen fraction was dried in a stove at 80° degrees. Approximately 5.5 mg of the obtained solid collagen was weighed in tin cups and combusted in an elemental analyzer-isotope ratio mass spectrometry (EA-IRMS) system. Collagen extraction and isotopic measurement took place at the Center for Isotope Research (CIO), University of Groningen.

The quality of the collagen was assessed through the atomic C:N ratio (C:N= (%C/12)/(%N/14)) as well as C and N percentage content following (116). Accepted values for the atomic C:N should be between 2.9 and 3.6, and for carbon and nitrogen content of the collagen (C%, N%), 30–45%, and 11–16%, respectively (116).

| **SCH BOS34** | | | **SCH BOS94** | | | **SCH BOS132** | | | **SCH BOS170** | | |
| --- | --- | --- | --- | --- | --- | --- | --- | --- | --- | --- | --- |
| **δ^13^C_VPDB COR_** | **δ^18^O_VPDB COR_** | **mm** | **δ^13^C_VPDB COR_** | **δ^18^O_VPDB COR_** | **mm** | **δ^13^C_VPDB COR_** | **δ^18^O_VPDB COR_** | **mm** | **δ^13^C_VPDB COR_** | **δ^18^O_VPDB COR_** | **mm** |
| -10.8 | -5.5 | 4.4 | -12.0 | -6.1 | 2.6 | -10.7 | -5.7 | 4.0 | -10.4 | -4.4 | 3.4 |
| -10.7 | -5.4 | 7.8 | -11.8 | -6.1 | 4.9 | -10.8 | -5.5 | 6.4 | -10.4 | -4.6 | 6.6 |
| -10.8 | -5.0 | 10.6 | -11.6 | -5.4 | 7.3 | -10.7 | -5.5 | 8.9 | -10.9 | -4.8 | 9.1 |
| -10.9 | -3.9 | 13.4 | -11.3 | -5.0 | 9.4 | -10.6 | -5.3 | 11.4 | -11.0 | -5.3 | 11.8 |
| -10.9 | -3.3 | 16.0 | -10.9 | -4.9 | 11.7 | -10.6 | -5.1 | 13.8 | -11.3 | -6.0 | 14.7 |
| -11.2 | -2.7 | 19.0 | -11.1 | -5.0 | 14.4 | -10.7 | -4.7 | 16.0 | -11.4 | -6.3 | 17.9 |
| -11.3 | -2.9 | 21.8 | -11.3 | -5.0 | 16.6 | -10.9 | -5.0 | 18.3 | -11.5 | -6.7 | 20.7 |
| -11.6 | -2.6 | 24.1 | -11.6 | -5.1 | 18.8 | -11.1 | -5.2 | 20.4 | -11.5 | -6.5 | 23.1 |
| -11.7 | -2.6 | 27.7 | -12.2 | -5.7 | 21.0 | -11.3 | -5.6 | 23.0 | -11.5 | -6.7 | 25.9 |
| -11.9 | -3.2 | 30.6 | -12.7 | -6.2 | 23.0 | -11.5 | -5.8 | 25.0 | -11.5 | -6.4 | 29.3 |
| -12.1 | -3.4 | 33.0 | -13.1 | -6.2 | 25.7 | -11.6 | -6.3 | 27.5 | -11.4 | -6.4 | 32.8 |
| -12.0 | -4.0 | 35.5 | -13.4 | -6.7 | 28.2 | -11.6 | -6.3 | 30.2 | -11.3 | -5.9 | 35.5 |
| -12.1 | -4.7 | 37.6 | -13.8 | -6.6 | 30.8 | -11.5 | -6.3 | 32.3 | -11.1 | -5.6 | 38.4 |
| -12.3 | -5.3 | 40.1 | -13.6 | -6.7 | 33.0 | -11.6 | -6.2 | 34.5 | -11.0 | -5.3 | 41.7 |
| -12.3 | -5.5 | 43.4 | -13.7 | -6.6 | 35.3 | -11.7 | -6.3 | 36.8 | -11.0 | -5.0 | 44.1 |
| -12.2 | -5.6 | 45.8 | -13.4 | -6.7 | 37.6 | -11.9 | -6.1 | 39.1 | -11.0 | -4.5 | 46.9 |
| -11.9 | -5.4 | 48.3 | -13.4 | -6.3 | 40.5 | -11.8 | -6.1 | 41.3 | -11.0 | -4.3 | 49.2 |
| -11.8 | -5.0 | 51.3 | -13.3 | -6.1 | 42.8 | -12.0 | -6.0 | 44.0 |  |  |  |
|  |  |  | -12.9 | -5.7 | 45.2 | -11.8 | -5.7 | 45.9 |  |  |  |
|  |  |  | -12.8 | -5.4 | 47.5 |  |  |  |  |  |  |
| **SCH BOS175** | | | **SCH BOS201** | | | **SCH BOS209** | | | **SCH BOS211** | | |
| **δ^13^C_VPDB COR_** | **δ^18^O_VPDB COR_** | **mm** | **δ^13^C_VPDB COR_** | **δ^18^O_VPDB COR_** | **mm** | **δ^13^C_VPDB COR_** | **δ^18^O_VPDB COR_** | **mm** | **δ^13^C_VPDB COR_** | **δ^18^O_VPDB COR_** | **mm** |
| -11.0 | ‒2.0 | 3.1 | -10.5 | -3.5 | 3.1 | -11.0 | -5.6 | 2.6 | -10.6 | -5.6 | 3.1 |
| -11.0 | ‒2.0 | 6.1 | -10.5 | -3.3 | 5.4 | -11.1 | -6.1 | 3.5 | -10.6 | -5.4 | 5.1 |
| -11.2 | -1.9 | 9.5 | -10.6 | -3.2 | 7.5 | -11.0 | -5.7 | 7.7 | -10.5 | -5.1 | 7.5 |
| -11.2 | -1.9 | 12.8 | -10.8 | -3.5 | 9.8 | -11.2 | -5.1 | 9.9 | -10.7 | -4.9 | 10.3 |
| -11.3 | -2.1 | 16.1 | -11.0 | -3.7 | 11.9 | -11.2 | -4.4 | 12.2 | -10.7 | -4.5 | 12.3 |
| -11.4 | -2.6 | 18.5 | -10.9 | -4.0 | 14.0 | -11.3 | -3.8 | 14.5 | -11.0 | -4.4 | 15.4 |
| -11.5 | -3.2 | 21.9 | -10.9 | -4.6 | 16.1 | -11.4 | -3.3 | 16.5 | -11.1 | -4.5 | 18.0 |
| -11.6 | -4.1 | 25.9 | -10.7 | -4.8 | 18.9 | -11.6 | -3.0 | 18.8 | -11.3 | -5.0 | 20.4 |
| -11.5 | -4.2 | 28.1 | -10.9 | -5.3 | 20.4 | -11.7 | -2.8 | 21.2 | -11.5 | -5.2 | 22.4 |
| -11.8 | -4.5 | 31.0 | -10.7 | -5.4 | 22.8 | -12.0 | -2.6 | 23.8 | -11.4 | -5.5 | 25.5 |
| -11.7 | -4.8 | 33.6 | -10.7 | -5.7 | 25.1 | -12.1 | -2.5 | 26.1 | -11.6 | -5.9 | 28.0 |
| -11.6 | -4.7 | 36.5 | -10.8 | -5.6 | 27.3 | -12.2 | -3.1 | 28.7 | -11.6 | -5.8 | 30.3 |
| -11.5 | -4.7 | 39.3 | -10.7 | -5.5 | 29.6 | -12.4 | -3.2 | 30.8 | -11.6 | -6.0 | 32.5 |
| -11.4 | -4.5 | 42.0 | -10.6 | -5.4 | 31.5 | -12.4 | -3.9 | 33.2 | -11.4 | -5.9 | 34.8 |
| -11.2 | -4.2 | 45.4 | -10.6 | -5.3 | 33.6 | -12.4 | -4.6 | 36.3 | -11.3 | -5.6 | 37.3 |
| -10.9 | -3.7 | 47.3 | -10.4 | -4.8 | 35.8 | -12.4 | -5.1 | 38.9 | -11.3 | -5.5 | 39.5 |
| -10.8 | -3.3 | 49.7 | -10.5 | -4.7 | 37.8 | -12.4 | -5.6 | 41.8 |  |  |  |
|  |  |  | -10.3 | -4.3 | 39.7 | -12.3 | -6.5 | 44.5 |  |  |  |
|  |  |  | -10.4 | -4.1 | 41.8 | -12.4 | -5.8 | 46.9 |  |  |  |
|  |  |  | -10.3 | -3.4 | 43.9 | -12.3 | -5.4 | 48.7 |  |  |  |
|  |  |  | -10.3 | -2.9 | 45.8 | -12.2 | -4.9 | 51.1 |  |  |  |
|  |  |  | -10.3 | -2.6 | 47.5 | -12.1 | -4.4 | 53.6 |  |  |  |

*S5 Table. Stable oxygen (δ^18^O) and carbon (δ^13^C) isotope values measured in cattle tooth enamel bioapatite from Schipluiden (mm = distance from the enamel–root junction).*

**References**

1. Kuitems M, van der Plicht J, Drucker DG, Van Kolfschoten T, Palstra SWL, Bocherens H. Carbon and nitrogen stable isotopes of well-preserved Middle Pleistocene bone collagen from Schöningen (Germany) and their paleoecological implications. J Hum Evol. 2015;89:105–13.

2. Longin R. New method of collagen extraction for radiocarbon dating. Nature. 1971;230:241–2.
